# Recommendations for the prevention and treatment of the novel coronavirus pneumonia in the elderly in China

**DOI:** 10.1002/agm2.12113

**Published:** 2020-06-09

**Authors:** Qiong Chen, Lijing Wang, Weiwei Yu, Huan Xi, Qiang Zhang, Xinyu Chen, Kui Huang, Xiang Lu, Xinmin Liu, CunTai Zhang, Jianye Wang, Chinese Geriatrics Society, National Center of Gerontology

**Affiliations:** ^1^ Department of Geriatrics National Clinical Research Center for Geriatric Disorders Xiangya Hospital Central South University Changsha China; ^2^ Department of Geriatrics Tongji Hospital Affiliated to The Tongji Medical College Huazhong University of Science and Technology Wuhan China; ^3^ National Center of Gerontology Beijing Hospital Beijing China; ^4^ Department of Geriatrics Tianjin Medical University General Hospital Tianjin Geriatrics Institute Tianjin China; ^5^ Department of Geriatrics Zhejiang Hospital Zhejiang Geriatric Medical Center Hangzhou China; ^6^ Department of Geriatrics Sir Run Run Hospital Nanjing Medical University Hubei Huangshi Jiangsu Novel Coronavirus Pneumonia Medical Team Nanjing China; ^7^ Department of Geriatrics First Hospital Peking University Beijing China

**Keywords:** 2019 novel coronavirus (2019‐nCoV), pneumonia, prevention and treatment, the elderly

## Abstract

The population is commonly susceptible to the 2019 novel coronavirus (2019‐nCoV), especially the elderly with comorbidities. Elderly patients infected with 2019‐nCoV tend to have higher rates of severe illness and mortality. Immunosenescence is an important cause of severe novel coronavirus pneumonia (NCP) in the elderly. Due to the combination of underlying diseases, elderly patients may exhibit atypical manifestations in clinical symptoms, supplementary examinations, and pulmonary imaging, deserving particular attention. The general condition of the elderly should be considered during diagnosis and treatment. In addition to routine care and measures—such as oxygen therapy, antiviral therapy, and respiratory support—treatment of underlying disease, nutritional support, sputum expectoration complication prevention, and psychological support should also be considered for elderly patients. Based on a literature review and expert panel discussion, we drafted the "Recommendations for the Prevention and Treatment of the Novel Coronavirus Pneumonia in the elderly in China," aiming to provide help with the prevention and treatment of NCP and the reduction of harm to the elderly population.

## 1 INTRODUCTION

In December 2019, there was an outbreak of novel coronavirus pneumonia (NCP) in Wuhan; the pathogen was 2019 novel coronavirus (2019‐nCoV), which had not previously been detected in the human body. The World Health Organization (WHO) officially named the disease “coronavirus disease 2019 (COVID‐19).” Meanwhile, the International Committee on Taxonomy of Viruses named the novel coronavirus “severe acute respiratory syndrome coronavirus 2 (SARS‐CoV‐2).” Due to the measures of preventive control and medical treatment, the rising momentum of the epidemic in China has been contained and the epidemic has been alleviated. However, morbidity abroad is on the rise and the virus is spreading globally. According to data from the currently released report, the pathogen may infect people of all ages, but the severe disease rate is higher among the elderly population, which accounts for the vast majority of mortalities.^1^, ^2^, ^3^ To curb the outbreak of novel coronavirus and reduce the harm to the elderly, we have compiled the “Key Points for the Prevention and Treatment of Novel Coronavirus Pneumonia in the Elderly (trial implementation)” in accordance with the recent literature.

## 2 ETIOLOGICAL FEATURES OF NOVEL CORONAVIRUS

On January 24, 2020, the Chinese Center for Disease Control and Prevention, National Institute for Viral Disease Control and Prevention published the virus seed information of “the first novel coronavirus,” in China, which shows that, like the 2003 severe acute respiratory syndrome coronavirus (SARS‐CoV) and the Middle East respiratory syndrome coronavirus (MERS‐CoV), the novel coronavirus belongs to the betacoronavirus genus. Gene sequence alignment shows that the novel coronavirus is most similar to the bat SARS‐like coronavirus (bat‐SL‐CoVZC45) with a homology of 88%; the equivalent rates for SARS‐CoV and MERS‐CoV are 79% and 50%, respectively.^4^

An essential receptor for SARS‐CoV is angiotensin‐converting enzyme 2 (ACE2) and this mainly results in the infection of cilia bronchial epithelial cells and type II alveolar epithelial cells. Studies^4^, ^5^ show that novel coronavirus is highly homologous to SARS‐CoV, which can be attributed to the combination of virus spike protein and human ACE2 receptor protein militated to the host. This receptor is mainly distributed in the pulmonary vascular endothelial cells, heart, kidney, and so forth. The physicochemical property data of the novel coronavirus mainly come from the study of SARS‐CoV. The virus is sensitive to ultraviolet light and heat. Exposure to 56°C for 30 minutes and lipid solvents, such as ether, 75% ethanol, chlorine‐containing disinfectant, peracetic acid, and chloroform, can effectively inactivate the virus. Chlorhexidine has not been effective in inactivating the virus.

## 3 EPIDEMIOLOGICAL FEATURES OF NCP AMONG THE ELDERLY

A total of 82 218 cases of NCP had been diagnosed in China up to March 28, 2020. Epidemiological studies^1^, ^2^, ^6^, ^7^, ^8^ show that at the early stage, the virus was transmitted to humans from wildlife, then from human to human, which caused an outbreak of infected people. Currently, patients infected by the novel coronavirus are the main source of infection; asymptomatic infected people can also be an infectious source. Transmission of the virus happens mainly through respiratory droplets and close contact. There is the possibility of aerosol transmission in a relatively closed environment with a long exposure to high concentrations of aerosol.^9^ The WHO estimated that the basic reproduction number (R_0_) of novel coronavirus is 1.4‐2.5.^10^ Based on early research from 425 patients, the estimated R_0_ is 2.2.^6^ A later study from 4021 patients showed that the estimated R_0_ was 3.77 (range, 2.23‐4.82), which shows that the transmissibility of novel coronavirus is equivalent to that of SARS‐CoV and higher than that of MERS‐CoV.^3^ Some studies also show that the transmissibility of novel coronavirus is higher than that of SARS‐CoV.^11^ Up to March 28, 2020, the cases of death from NCP had reached 3301 with a case fatality rate of 4.0%. According to the available data, the mortality rate of NCP is lower than that of SARS‐CoV (9.6%) and MERS‐CoV (34%).^12^

People are commonly susceptible to 2019‐nCoV, especially the elderly with comorbidities, such as diabetes, hypertension, cardiovascular disease, and cerebrovascular disease.^1^, ^2^ The elderly are more likely to have severe cases and to require intensive care unit (ICU) care,^2^, ^3^ and this population has a high mortality rate. A study^13^ of 1099 patients diagnosed with 2019‐nCoV showed that the median age of patients was 47.0 years with 15.1% of all patients and 27% of severely ill patients aged over 65 years, and a median incubation period of 3.0 days (range, 0 to 24 days). An analysis from the research of 4021 diagnosed patients showed that nearly half (47%) of the patients were aged over 50 years and 1052 patients (26.2%) were aged over 60 years. The mortality of patients (some with complications) aged over 60 years (5.3%) was significantly higher than that of those aged under 60 years (5.3%).^3^ The Chinese Center for Disease Control and Prevention has analyzed all cases of patients with coronavirus pneumonia in the Chinese Reporting System for Infectious Diseases. Up to February 11, 2020, there were 44 672 confirmed cases, in which the proportions of patients aged over 60 were 44.1% in Wuhan, 35.1% in Hubei province (including Wuhan), and 31.2% in China (including Hubei).^14^

## 4 POSSIBLE MECHANISM OF NCP IN THE ELDERLY WITH HIGH RISK OF INFECTION

A major reason for the widespread transmission of the novel coronavirus in the population is the lack of immunocompetence, and there is no specific medicine for this virus. In healthy young people with normal immunity, the virus can be effectively eliminated by rapidly mobilizing the body’s immune function.

There are many age‐related variations in the respiratory and immune systems.^15^, ^16^ As for the elderly, there are many variations in the respiratory system’s physiological function caused by the change of pulmonary anatomical structure and muscle atrophy, such as the weakening of airway clearance, the reduction of lung reserve, and the depression of defensive barrier function. In addition, pro‐inflammatory cytokines baselines in the tissue and circulation of the elderly rise with age, especially interleukin (IL)‐1β, IL‐6, and tumor necrosis factor‐α (TNF‐α), resulting in a condition known as “inflammatory senescence.” Corresponding to inflammatory senescence, the body's immune response to pathogenic threaten or tissue damage is blunt, called "Immunosenescence." With age, the function of innate immunity and adaptive immunity declines; the discernment, chemotaxis, and phagocytosis of macrophages, natural killer cells, and neutrophils downgrade; and the diversity of the T‐cell receptor (TCR) decreases. Furthermore, at the age of 60 years, the thymus that produces immature T‐cells is replaced by adipose tissue, which leads to the accumulation of memory T‐cells and effector T‐cells and results in the decrease of immature T‐cells. Meanwhile, the B‐cells’ ability to produce antibodies declines with age, which may cause hypoimmunity.

Inflammatory senescence and immunosenescence make individuals susceptible to novel coronavirus. The elderly in morbid states, such as chronic obstructive pulmonary disease or extra‐pulmonary organ system diseases, are more likely to have severe respiratory infections, which causes the high mortality in elderly patients with NCP. The specific mechanism by which inflammatory factor storms are more likely to develop into acute respiratory failure in the elderly infected with novel coronavirus is unknown. It is still to be studied whether this mechanism is associated with the inflammatory imbalance that leads to disruption of pulmonary immune homeostasis and/or the decline of innate immunity and adaptive immunity that aggravate novel coronavirus transmission, which leads to the imbalance response of pro‐inflammatory and anti‐inflammatory cytokines.^17^

The novel coronavirus mainly leads to pulmonary infection, which increases the heart’s workload, causes hyperglycemia, and makes it harder to control the infection. The characteristics of multisystem disease in the elderly make it a complex condition with all the diseases interplaying with each other, which increases the difficulty of treatment.

## 5 CLINICAL MANIFESTATION OF NCP

Based on a current epidemiological survey, the incubation of NCP is 1‐14 days, and mostly 3‐7 days. The most common symptoms are fever, dry cough, and weakness. Nasal congestion, runny nose, sore throat, emesis, and diarrhea are found in some cases. Severe patients develop dyspnea and/or hypoxemia after 1 week and may progress rapidly to acute respiratory distress syndrome (ARDS), septic shock, refractory metabolic acidosis, coagulopathy, multiple organ failure, and so forth. There are no clinical data on novel coronavirus infection in the elderly. However, the clinical symptoms of pneumonia in elderly patients are often atypical, so if elderly patients have dyspnea, ARDS, or unexplained systemic symptoms, such as fatigue, shock, coagulation disorders, or other symptoms, the possibility of new coronavirus infection should be considered.

With the popularization of etiological examination, many suspected patients with mild cases have been diagnosed. However, there are neither clinical studies about the clinical characteristics of these mild patients, nor clinical studies about the proportion and characteristics of the elderly. Nevertheless, they are still significant in the prevention and control of disease transmission, and patients with atypical manifestations cannot be ignored.

## 6 LABORATORY TEST AND CHEST IMAGING FOR NCP

In the early stages of the disease, peripheral white blood cell count is normal or decreased and the lymphocyte count is decreased. Some patients have elevated liver enzymes, muscle enzymes, and myoglobin. Most patients have elevated C‐reactive protein and erythrocyte sedimentation rate and normal procalcitonin. The levels of some inflammatory cytokines, such as IL‐2, TNF‐α, IL‐6, and interferon‐γ, are normal or higher. In severe cases, D‐dimer increases and peripheral blood lymphocytes progressively decrease.

In the early stage, chest imaging shows multiple small patchy shadows and interstitial changes, which are more apparent in the peripheral zone of the lungs. As the disease progresses, imaging shows multiple ground‐glass opacities and infiltration in both lungs. In severe cases, pulmonary consolidation may occur. However, pleural effusion is rare. The elderly often suffer from chronic diseases that may effect and disturb the auxiliary examinations, which should be considered minutely in the process of treatment. For example, the elderly may have an increase in blood parameters and procalcitonin earlier than other patients, which is caused by co‐bacterial infection; they may experience patient delay caused by atypical symptoms, which makes the initial manifestation of laboratory examination atypical; and underlying pulmonary diseases may lead to the early manifestation of atypical pulmonary imaging and, under these circumstances, the dynamic observation of past imaging data and imaging features is more meaningful.

## 7 DIAGNOSTIC CRITERIA FOR NCP

We recommend the adoption of the criteria in the “Diagnosis and Treatment Protocol for Novel Coronavirus Pneumonia (trial version 7).”^9^ The details are as follows:

1. The suspected case: correspond with any article in epidemiology history; correspond with any two articles in clinical manifestation; and correspond with any three articles in clinical manifestation without definite epidemiology history.
  - Epidemiology history: (a) history of travel or residence in Wuhan City and its surrounding areas or other case‐reported communities within 14 days before the onset of the disease; (b) contact history with novel coronavirus‐infected person (positive in pathogen nucleic acid detection) within 14 days before onset; (c) contact history with patients who had fever or respiratory symptoms from Wuhan City and its surrounding areas or a community with case reports within 14 days before onset; and (d) clustered onset (two or more cases of fever and/or respiratory symptoms in small spaces, such as home, office, classroom, etc, within 2 weeks).
  - Clinical manifestation: (a) fever and/or respiratory symptoms; (b) imaging features of NCP: patchy shadows and pulmonary interstitial changes at the early stage with obvious lung periphery and further bilateral pulmonary multiple ground‐glass opacity and infiltration, severe patients may have consolidation of the lung, pleural effusion or (infrequently) lymphadenectasis; (c) in the early stage of onset, the total number of white blood cells is normal or decreases, or the lymphocyte count decreases.
2. The confirmed case: based on the criteria of the suspected case, with one of the following etiological or serological factors:
  - Positive in novel coronavirus nucleic acid detection by real‐time fluorescence reverse transcription polymerase chain reaction.
  - The genetic sequence of virus has a high homology with known novel coronavirus.
  - With positive specific IgM and IgG antibodies of novel coronavirus in serum, the specific IgG antibodies of novel coronavirus in serum increased from negative to positive or recovered four times higher than those in acute phase.

## 8 CLINICAL CLASSIFICATION OF NCP

Clinical classifications should be made according to the “Diagnosis and Treatment Protocol for Novel Coronavirus Pneumonia (trial version 7)”^9^ published by the National Health Commission and the National Administration of Traditional Chinese Medicine:

- Mild case: with mild clinical symptoms, no pneumonia in imaging.
- Ordinary case: with fever, respiratory, and other symptoms, with pneumonia manifestation in imaging.
- Severe case: corresponds with any of the following: (a) respiratory distress (respiratory rate ≥ 30 times/min); (b) oxygen saturation ≤ 93% at resting state; or (c) arterial partial pressure of oxygen/fraction of inspired oxygen ≤ 300 mm Hg (1 mm Hg = 0.133 kPa). Cases with chest imaging that shows obvious lesion progression within 24‐48 hours of >50% should be managed as severe cases.
- Critical case: corresponds with any of the following: (a) respiratory failure and requiring mechanical ventilation; (b) shock; or (c) other organ failure that requires ICU care.

## 9 DIFFERENTIAL DIAGNOSIS OF NCP

NCP should be distinguished from influenza virus, parainfluenza virus, adenovirus, respiratory syncytial virus, and any other known respiratory virus that can cause upper respiratory infection and pneumonia; it should be distinguished from bacterial pneumonia, mycoplasma, and chlamydia pneumonia; and it should be distinguished from nodular vasculitis, multiple myositis, and organized pneumonia.

## 10 SPECIAL PROBLEMS AMONG ELDERLY PATIENTS DURING DIAGNOSIS OF NCP

For elderly patients, clinical typing must be accompanied by prospective judgment, such as the consideration of functional status of other systems, as lung lesions of elderly patients may be more likely to induce failure in other systems, which may even occur before respiratory failure. It is advisable to conduct a comprehensive assessment of elderly patients. By doing so, elderly high‐risk patients who may tend to get severe/critical illness will be identified and provided intervention as soon as possible, which can improve the prognosis.

## 11 MEASURES OF RESPONSE AND TREATMENT FOR NCP

### 11.1 Case finding and reporting

Once medical personnel at all levels and all types of institutions find suspected cases that meet the definition, they should immediately carry out single‐person isolation treatment. After consultation of in‐hospital experts or attending physicians, those who are still considered as suspected cases should be reported through direct network within 2 hours and led to the collection of specimens for virus nucleic acid testing, meanwhile transporting patients to designated hospital as soon as possible with safety ensured. Even patients with common respiratory pathogens who have had close contact with positive novel coronavirus patients are advised to take a timely novel coronavirus nucleic acid test. Suspected cases can be excluded if the respiratory pathogens in nucleic acid testing are negative (sampling interval must be at least 24 hours) and the IgM and IgG of the novel coronavirus‐specific antibodies are still negative 7 days after onset.

### 11.2 Treatment venue determined by the severity of the disease

Suspected and confirmed cases should be treated in a designated hospital with effective isolation and protection; suspected cases should receive isolated treatment in one single room separately while confirmed cases can be accommodated in the same room. Critical cases should be treated in the ICU as early as possible.

### 11.3 Treatment

#### 11.3.1 Hospitalization

- General treatment: Allow patients to rest in bed and provide strengthening support therapy; ensure sufficient caloric intake for patients; monitor patients’ water, electrolyte, and acid‐base balances to maintain internal environment stability; and closely monitor vital signs and oxygen saturation.
- Sputum drainage: The sputum‐drainage ability of elderly patients can decrease and sometimes they need mechanical assistance. Assisted sputum drainage procedures should be strictly followed.
- Oxygen therapy: Patients with hypoxemia should be provided with oxygen therapy immediately, with blood oxygen saturation maintaining at ≥90%. Note: (1) Oxygen therapy methods: Patients with mild symptoms should receive normal nasal catheter and mask oxygen supply; patients with severe symptoms suffering from increased respiratory distress or where standard oxygen therapy has had no effects can be given high‐flow nasal cannula (HFNC) oxygen therapy, starting at 20 L/min and gradually increased to 50‐60 L/min. In the meantime, adjust oxygen concentration (fraction of inspired oxygen) according to the oxygenation targets. For patients with chronic obstructive pulmonary disease, the decision of whether they need to use HFNC oxygen therapy should be made after assessing the possible risk of carbon dioxide retention. If possible, inhalation of mixed hydrogen and oxygen can be applied. (2) Respiratory support methods: The non‐invasive ventilator, which is not recommended prior to HFNC oxygen therapy, should be adopted immediately once the HFNC oxygen therapy fails to reach the treatment objectives as desired. The non‐invasive ventilator is the preferred mechanical ventilation mode if the medical staff can achieve complete protection. If non‐invasive mechanical ventilation works for 2 hours showing no improvements on patients, or patients cannot tolerate non‐invasive ventilation with increased airway secretion, severe cough, or unstable hemodynamic, close observation should be arranged and, if necessary, invasive ventilation can be used. For invasive mechanical ventilation, use the “ventilation strategy for lung protection,” low tidal volume (4‐8 mL/kg predicted bodyweight) and low inspiratory pressure (platform pressure < 30 cm H_2_O; 1 cm H_2_O = 0.098 kPa) to reduce ventilator‐related lung injury. Closed sputum suction is selected according to the condition of airway secretion. If necessary, bronchoscopy should be used with appropriate treatment. When it comes to the most serious time, prone‐position ventilation, lung recruitment, or extracorporeal membrane oxygenation can be used. However, as regards treating critically ill patients, the main recommendation of mechanical ventilation among the elderly is the non‐invasive ventilator. Non‐invasive mechanical ventilation should be used carefully after full evaluation of benefits.
- Antiviral therapy: It is advised to try α‐interferon aerosolized inhalation (5 million units each time, 2 mL, 2 times/d with sterilized injection water), lopinavir/ritonavir (two capsules, 2 times/d, no more than 10 days per session), umifenovir (200 mg, 3 times per day, no more than 10 days of treatment), or ribavirin intravenous injection (500 mg, 2‐3 times/d, no more than 10 days per session) additionally. Hospitals can try α‐interferon (5 million U or equivalent dose each time, adding 2 mL of sterilized water, atomization inhalation twice daily), lopinavir/ritonavir (200 mg/50 mg per pill, two pills each time, twice daily, no longer than 10 days), ribavirin (suggested to be used jointly with interferon or lopinavir/ritonavir, 500 mg each time for adults, twice or three times of intravenous injection daily, no longer than 10 days), chloroquine phosphate (for the elderly aged ≤ 65 years, 500 mg bid for 7 days with bodyweight over 50 kg; 500 mg bid for Days 1‐2 and 500 mg qd for Days 3‐7 with bodyweight below 50 kg [note: chloroquine cannot be used for patients with heart diseases]), and umifenovir (200 mg tid, no longer than 10 days). The clinical effect of the above drugs in the treatment of NCP still lacks large‐scale clinical research and evidence‐based medical evidence. In clinical application, the efficacy of all currently tested drugs needs to be further evaluated. Since the elderly have poor tolerance to medicine, with different diseases, they often take several kinds of medicine, which may result in adverse reactions, such as liver and kidney damage. Antiviral drugs should be used cautiously. During the treatment process, adverse reactions should be monitored. If there are intolerable adverse reactions, the drug should be stopped in time and symptomatic treatment should be given.
- Glucocorticoid: Severe patients can take glucocorticoids at a dose of 1‐2 mg/kg/d, and the recommended course of treatment is 3‐5 days.
- Antibiotics: Blind or improper use of antimicrobial medicine, especially combined use of broad‐spectrum antibacterial medicine, should be avoided. Strengthen bacteriological monitoring and apply antimicrobial medicine immediately when there is evidence of secondary bacterial infection.
- Circulation support: Improve microcirculation and use vasoactive medicine on the basis of adequate fluid resuscitation; if necessary, take the hemodynamic monitoring for ARDS patients with conservative volume control when hypoperfusion is ensured.
- Intestinal microecological regulator can be used to maintain intestinal microecological balance.
- As elderly patients are prone to multiple system organ dysfunction and even failure, other systemic complications should be prevented, including disseminated intravascular coagulation and deep vein thrombosis, delirium and double infection occurring in gastrointestinal hemorrhage, renal failure, and coagulation disorders.
- Psychological support should be given to patients and their families. Support measures can be carried out in accordance with the guiding measures of “Guiding Principles of Urgent Intervention Psychological Crisis in Novel Coronavirus Pneumonia Outbreak” issued by Disease Prevention and Control Bureau of National Health and Health Commission on January 1, 2020.^18^
- It is not advocated to use human immunoglobulin routinely. Human immunoglobulin is a kind of passive immunity and no novel coronavirus infection has occurred before, with no related antibody, so human immunoglobulin should not be taken as routine use. If elderly patients still have low levels of immunoglobulin after testing, human immunoglobulin can be used as appropriate. In severe cases, gamma globulin can be administered intravenously as appropriate.
- For severe patients with low lymphocyte count and low cellular immune function, we recommend the use of thymosin α1.
- For patients with severe cases, such as extensive lesions of both lungs, and with increasing levels of IL‐6 in laboratory testing, tocilizumab treatment can be tried.
- For severe and critical patients with “cytokine storm,” for the purpose of clearing inflammatory cytokines and blocking the cytokine storm, blood purification treatment can be used.
- Traditional Chinese Medicine treatment: this disease is in the category of TCM epidemic. TCM treatment based on syndrome differentiation can be carried out according to the “Novel Coronavirus Pneumonia Diagnosis and Treatment Plan (trial version 7).”^9^
- For patients with fast progression, severe cases, or critical cases, the plasma treatment of rehabilitated patients can be used as appropriate. Usage and dosage should refer to “Protocol of Clinical Treatment with Convalescent Plasma for COVID‐19 Patients (2nd trial version).”^19^

#### 11.3.2 Home‐based care

Suspected cases can choose home‐based care as appropriate after the infection of novel coronavirus has resolved; patients who have been diagnosed with novel coronavirus and are recovered fully are advised to continue 14‐day isolated home‐based care and health monitoring. As the elderly often suffer from several diseases with reduced ability of daily life, attention should also be paid to whether the follow‐up medical system is complete after home‐based care, which should ensure patients’ medical security and provide medical services if there are any changes in patients’ conditions.

In home‐based care, the elderly should be very careful of nutritional balance. Malnutrition is one of the most important negative factors affecting the prognosis of disease among elderly patients. The elderly are recommended to have a balanced diet and balanced intake of calories, protein, vitamins, minerals and so on, with meat and vegetables to ensure adequate nutrition. Elderly people should eat foods that are easily digested, eat more vegetables and fruits, drink water frequently, and avoid eating wild animals and rotten or expired food. Chilled poultry should be purchased through regular channels, and meat, eggs, and milk should be fully cooked before eating. Elderly people with poor appetite and eating inadequate food can take some protein and trace elements appropriately through nutritionally fortified foods, special medical formula foods, or nutrient supplements. For all elderly people, attention should also be paid to avoid aspiration pneumonia due to improper inhaling. Patients and their families nursing at home should receive standardized health education and training, including room arrangements, hand hygiene, proper use of masks, respiratory hygiene, disinfection standard of household articles, contact information of medical institutions, nutrition and health knowledge, psychological self‐regulation skills, and disease‐monitoring methods. After confirmation of home‐based care with patients, the medical staff should assess the patient’s general condition and reconfirm the patient’s medical treatment in the past and home‐based medical treatment plan, and make sure patients and their families understand those. Recovered patients with isolated home‐based care are also suggested to have follow‐up to the hospital at 2 and 4 weeks post‐recovery.

At the same time, medical personnel should also inform the patients of which indications require contact immediately for consultation and hospital treatment. If one of the following situations occurs, home‐based care should be considered for reevaluation or even suspension: increased dyspnea; (fever patients unable to bring their temperature below 37°C after using antipyretic analgesics according to the instructions or still relying on the antipyretic and analgesic medicine to control fever after 3 days; (c patients suffer from purulent sputum, hemoptysis, or other systemic manifestations, such as chest pain, nausea, vomiting, diarrhea, decreased urine volume, mental disorders, unexplained decrease in blood pressure (systolic blood pressure more than 20 mm Hg lower than daily values); or family members or nursing staff in close contact are newly diagnosed with novel coronavirus infection or other suspected infections.

### 11.4 Protection during transportation

- Severe patients requiring tracheal intubation should be immediately transferred to the ICU with negative pressure conditions for treatment once diagnosed as requiring tracheal intubation. The operation should be carried out in accordance with the requirements of comprehensive protection.
- During transit, an oxygen storage mask should be used to provide oxygen of >15 L/min, in order to ensure oxygen storage airbag inflation is satisfied.
- Tracheal intubation should be induced with a standard and rapid sequence, with muscle relaxants used as far as possible to avoid the spread of droplets caused by coughing.
- After intubation, the eye mask and other reusable items should be sterilized with Jianzhisu (compound alcohol disinfectant) before removal from the negative pressure ward.
- The closed sputum suction should be used for patients with intubation in order to avoid air transmission caused by the ventilator’s airflow.
- In special cases, when the ventilator must be cut off for airway operation, the standby function of the ventilator should be used to avoid airborne transmission caused by the ventilator’s airflow; if the ventilator has no standby function, the Y‐type nozzle should be blocked to avoid airborne transmission.

### 11.5 Criteria for being discharged from hospital

Patients with a temperature returning to normal for more than 3 days, significantly improved respiratory symptoms, significantly absorbed pulmonary imaging inflammation, and respiratory pathogens nucleic acid testing showing negative for two consecutive times (sampling interval must be at least 24 hours) can be released from isolation or transferred to the appropriate department for treatment of other diseases.

## CONFLICTS OF INTEREST

Nothing to disclose.

## APPENDIX A

Members of the Experts Committee (in the sequential order of family name by pinyin): Linzi Deng (Beijing Hospital, National Center of Gerontology), Xinglin Gao (Respiratory Department of Guangdong Provincial People's Hospital in East District, Guangdong Academy of Medical Sciences, Guangdong Geriatrics Institute), Ping Lei (Department of Geriatrics, Tianjin Medical University General Hospital, Tianjin Geriatrics Institute), Liuyi Li (Department of Infection Management , First Hospital, Peking University), Xiaohong Liu (Department of Geriatrics, Peking Union Medical College Hospital), Youshuo Liu (Department of Geriatrics, the Second Xiangya Hospital, Central South University), Yongjun Mao (Department of Geriatrics, the Affiliated Hospital of Qingdao University), Li Sun (Department of Geriatric Respiratory, Shanxi Provincial People’s Hospital), Xiping Tuo (Department of Geriatric, Changhai Hospital, Second Military Medical University), Jianqing Wu (Department of Geriatrics, the First Affiliated Hospital of Nanjing Medical University), Jing Yan (Department of Geriatrics, Zhejiang Hospital), Yunmei Yang (Department of Geriatrics, the First Affiliated Hospital of Zhejiang University Medical College), Pulin Yu (Beijing Hospital, National Center of Gerontology), Qin Zhang (Department of Geriatrics, the First Affiliated Hospital of Zhejiang University Medical College), Baiyu Zhou ( Beijing Hospital, National Center of Gerontology).
